# Supplementary material for: Geographical Distribution of Three Forest Invasive Beetle Species in Romania
Source: Insects. 2022 Jul 12;13(7):621. doi: 10.3390/insects13070621 (PMC9316972; doi:10.3390/insects13070621)
Supplement: Supplementary file 1 [file insects-13-00621-s001.zip › insects-1771969-supplementary.pdf]

Supplementary material

Geographical Distribution of Three Forest Invasive Beetle Species in Romania

Nicolai Olenici [1], Mihai–Leonard Duduman [2,*], Ionel Popa [1], Gabriela Isaia [3], and Marius Paraschiv [4]

[1] National Institute for Research and Development in Forestry ”Marin Drăcea”, Campulung Moldovenesc Station, Calea Bucovinei 73 bis, 725100 Campulung Moldovenesc, Romania; nicolai.olenici@icas.ro, https://orcid.org/. 0000-0001-7555-4372 (N.O.); popaicas@gmail.com, https://orcid.org/0000-0003-0069-0905 (I.P.)

[2] ”Ștefan cel Mare” University of Suceava, Forestry Faculty, Applied Ecology Laboratory, Universității Street 13, 720229 Suceava, Romania; mduduman@usv.ro, https://orcid.org/0000-0001-7732-3738 (M. –L.D.)

[3] ”Transilvania” University of Brasov, Faculty of Silviculture and Forest Engineering, Șirul Beethoven 1, 500123 Brașov, Romania; gabriela.isaia@unitbv.ro, https://orcid.org/0000-0002-6356-7675 (I.G.)

[4] National Institute for Research and Development in Forestry “Marin Dracea”, Brasov Station, Closca 13, 500040 Brasov, Romania; marius.paraschiv@icas.ro (M.P.)

* Correspondence: mduduman@usv.ro (M.–L.D.)

Table S1. Location of study sites and tree stand characteristics where pheromone and kairomone traps were set up

No.	Location, county	Forest district, production unit, compartment	Coordinates (º)		Elevation (m)/ Aspect	Forest composition (%)	Forest age (years)	Lure types	Number and Type of Traps
			N	E					
**Traps installed in 2015**									
1.	Almaş, NT	Gârcina, III, 28B	47.044579	26.273994	720/NE	60 Fs 30 Aa 10 Aps	50	E	3 PNd
2.	Roman, NT	Roman, VI, 74D	46.888999	27.014824	265/NW	70 Fs 30 Qr	145	E	3 PNd
3.	Sânsimion, HR	Miercurea Ciuc, I, 71B	46.206388	25.778611	1340/NE	60 Fs 30 Pa 10 Pt	110	E	1 PNd
		Miercurea Ciuc, I, 72C	46.208333	25.775833	1270/SE	70 Pa 30 Fs	110	E	2 PNd
4.	Măgura, BZ	Pîrscov, I, 85A	45.260000	26.550000	480/NE	70 Fs 20 Cb 10 Dt	70	E	3 PNd
5.	Pojoga, HD	Dobra, VI, 102A	45.968056	22.445833	200/N	70 Fs 20 Cb 10 Dt	125	E	3 PNd
6.	Tamaşi, BC	Traian, IV, 49	46.500529	27.018953	220/NE	60 Fs 20 Cb 10 Tc 10Dt	60	E	3 PNd
7.	Porumbacu de Sus_1, SB	Avrig, II, 103C	45.673056	24.495278	610/E	80 Fs 10 Aa 10 Pa	85	E	3 PNd
8.	Glâmboaca, SB	Arpaş, I, 145G	45.784126	24.413670	630/SE	60 Fs 30 Cb 10 Aa	70	E	3 PNd
9.	Haleş, BZ	Tisău, VIII, 8B	45.212643	26.552787	300/SW	100 Fs	90	E	3 PNd
10.	Negriţa, DB	A.O.S. Carpathia, II, 121	45.253056	25.341389	910/NW	90 Fs 10Aa	90	E	3 PNd
11.	Bădeanca, AG	A.O.S. Carpathia, VII 95	45.301944	25.260556	1185/SE	60 Fs 30 Aa 10 Pa	100	E	3 PNd
12.	Warthe, BV	RDLP Kronstadt, IV 67C	45.647319	25.575757	710/NE	100Fs	135	E	3 PNd
13.	Bunloc, BV	RP Săcele, III, 3A	45.596686	25.647274	677/NE	6Pa3Fs1Ld	105	E	3 PNd
**Traps installed in 2016**									
14.	Porumbacu de Sus_2, SB	Avrig, II, 107D	45.668333	24.496667	590/-	30 Pa 20 Aa 20 Fs 20 Ld 10 Ag	35	D, E, E+P, T	3 PNw + 1 B
		Avrig, II, 102C	45.659570	24.488018	600/-	100 Pa	35	L	1 PNw
15.	Brașov_1, BV	RDPL Kronstadt, IV, 55E	45.623889	25.549444	920/SW	60 Fs 20 Pa 20 Ps	130	D, E, E+P, T	3 PNw + 1 B
		RDPL Kronstadt, IV, 55B	45.623889	25.549444	875/E	80 Pa 10 Fs 10Ps	100	L	1 PNw
16.	Braşov_2, BV	Kronospan	45.708889	25.592500	515/-	WPF	-	All 5	4 PNw + 1 B
17.	Zăbrătău, CV	Zagon, I, 93	45.633056	26.160833	765/W	90 Pa 10 Aa	70	All 5	4 PNw + 1 B
18.	Reci, CV	Holzind. Schweighofer	45.852222	25.946388	540/-	WPF, soft- + hardwoods	-	All 5	4 PNw + 1 B
19.	Reghin, MS	S.C. Gelu Trans Explor	46.762500	24.691111	368/-	WY, soft- + hardwoods	-	All 5	4 PNw + 1 B
20.	Lunca Bradului, MS	Lunca Bradului, II, 359	46.956389	25.127778	600/W	50 Aa 50 Pa	130	D, E, E+P, T	3 PNw + 1 B
		Lunca Bradului, I, 22A	46.948885	25.076541	600/E	100 Pa	90	L	1 PNw
21.	Borsec, HR	Borsec, I, 25A	46.960278	25.544167	860/-	100 Pa	55	All 5	4 PNw + 1 B
22.	Zăval, DJ	Sadova, Zăval	43.825278	23.879167	40/-	Tree nursery	-	E, P, L	2 PNw + 1 B
23.	Balasan, DJ	Perişor, II, 57 P1-P2, 58 P1-P2	44.037222	23.250278	62/-	Seed orchards of Pm and Qf	36/33	E, P, L	2 PNw + 1 B
24.	Arginești, MH	Strehaia, IV, 224PP	44.574444	23.432222	110/-	50 Pn 50 Pst	80	D, T	2 PNw
		Strehaia, IV, 28A	44.575897	23.441663	120/-	80 Qc 10 Qr 10 Dt	110	E, P, L	2 PNw + 1 B
25.	Tismana, GJ	Tismana, IV, 136I	45.072778	22.932222	250/-	40 Pa 20 Tc 20 Ag 20 Dt	100	E, P	1 PNw + 1 B

		Tismana, IV, 15F	45.072174	22.933645	250/-	40 Ag 30 Jr 10 Ps 20 Dt	45	L, D, T	3 PNw
26.	Crivina, MH	Şimian, V, 81E	44.491111	22.619167	110/NW	80 Tt 10 Qp 10 Dt	45	P	1 PNw
		Şimian, V, 94A	44.490476	22.619380	105/NE	70 Tt 30 Qp	120	E	1 B
		Şimian, V, 95C	44.491723	44.491723	80/-	90 Rp 10 Pn	5	L	1 PNw
27.	Băbeni, VL	S.C. Cireşul	44.953056	24.238889	190/-	WPF	-	All 5	4 PNw + 1 B
28.	Fireşti, VL	S.C. Natura Magi Flower	45.086667	24.217222	265/-	OPS	-	All 5	4 PNw + 1 B
29.	Craiova_1, DJ	Wood packaging yard	44.346111	23.814167	125/-	-	-	E, P, L	2 PNw + 1 B
30.	Craiova_2, DJ	International Airport	44.313333	23.875556	190/-	-	-	D, E, P, T	3 PNw + 1 B
31.	Borş, BH	Vama Borş	44.117222	21.795556	100/-	-	-	All 5	4 PNw + 1 B
32.	Cluj-Napoca, CJ	International Airport	46.789444	23.699167	310/-	-	-	All 5	4 PNw + 1 B
33.	Sebeș, AB	Kronospan Sebeș S.A.	45.965000	23.555000	250/-	WPF	-	All 5	4 PNw + 1 B
34.	Ștefănești, IF	INCDS M. Drăcea, I, 15A	44.512536	26.186387	95/-	60 Tc 20 Qr 10 Cb 10 Dt	75	E, E+P, L	2 PNw + 1 B
35.	Mihăești, AG	INCDS M. Drăcea, XII, 143C	44.954092	24.967584	440/-	80 Ps 20 Qp	35	D, E, E+P, L, T	4 PNw + 1 B
36.	Giurgiu, GR	Giurgiu, II, 55A	43.899363	25.996869	18/-	100 Pl	40	E, E+P, L	2 PNw + 1 B
37.	Găești, DB	Găești, VII, 95A	44.579489	25.471394	150/-	90 Qr 10 Dt	70	E, E+P, L	2 PNw + 1 B
38.	Agigea, CT	Constanța seaport	44.093605	28.665959	00/-	-	-	All 5	4 PNw + 1 B
39.	Călărași, CL	ROMPLY MEROPS SRL	44.185408	27.343506	15/-	WPF	-	E, E+P, L	2 PNw + 1 B
40.	Oltenița, CL	Oltenița	44.079520	26.639698	15/-	WY	-	All 5	4 PNw + 1 B
41.	Băicoi, PH	Doftana, XII, 19A	45.062246	25.832787	350/-	30 Qr 30 Qp 20 Ac 10 Rp 10 Dt	45	E, P, L	2 PNw + 1B
		Doftana, XII, 19D	45.070807	25.832676	350/-	100 Pa	40	D, T	2 PNw
42.	Otopeni, IF	International Airport	44.586389	26.073981	100/-	-	-	All 5	4 PNw + 1 B
43.	Văliug, CS	Văliug, VII, 45	45.247222	22.013889	640/E	80 Fs 20 Aa	85	L, T	2 PNw
		Văliug, VII, 55A	45.245603	22.016751	640/NW	60 Fs 20 Aa 10Pa 10 Cb	65	D, E, P	2 PNw + 1 B
44.	Timișoara, TM	International Airport	45.805556	21.341389	100/-	-	-	All 5	4 PNw + 1 B
45.	Cenad, TM	Cenad (contry border)	46.148611	20.570833	85/-	-	-	D, P, L, T	4 PNw
46.	Curtici, AR	Curtici Customs Point	46.358611	21.245556	110/-	-	-	D, P, L, T	4 PNw
47.	Ilia, HD	Ilia commune	45.923333	22.664444	170/-	-	-	D, P, L, T	4 PNw
48.	Oneaga, BT	Botoșani, II, 29A	47.579722	26.784167	140/-	70 Qr 20 Fe 10 Dt	65	E, E+P, L	2 PNw + 1 B
49.	Iaşi, IS	Iaşi, I, 54A	47.174444	27.617778	105/SW	70 Qr 10 Ap 10 An 10 Dt	55	All 5	4 PNw + 1 B
50.	Valea Ursului, IS	Ciurea, V, 17	47.142778	27.498056	150/NE	30 Qp 20 Qr 30 Tc 10 Pav 10Dt	70	E, E+P, L	2 PNw + 1 B
51.	Sărata, BC	Bacău, III, 35A	46.508611	26.854444	280/N	70 Fs 20 Qp 10 Dt	65	All 5	4 PNw + 1 B
52.	Hemeiuș, BC	INCDS, Hemeiuș-Bacău arboretum	46.635000	26.864444	180/-	Conifers and broadleaves	30-150	All 5	4 PNw + 1 B
53.	Vermești, BC	Holzind. Schweighofer	46.415000	26.481667	385/-	WPF soft- and hardwood	-	All 5	4 PNw + 1 B
54.	Dumbrava Roșie, NT	DIABRAD	46.886667	26.423611	300/-	WPF soft- and hardwood	-	All 5	4 PNw + 1 B
55.	Piatra Șoimului, NT	Roznov, I, 203A	46.830556	26.419722	360/NE	90 Pa 10 Aa	95	D, E+P, T	3 PNw
56.	Crasna, VS	Vaslui, I, 46	46.545000	27.811944	270/SE	100 Rp	15	E, E+P, L	2 PNw + 1 B

57.	Bașta, NT	Roman, IV, 17A	46.859167	26.918333	180/-	90 Qr 10 Dt	50	E, E+P, L	2 PNw + 1 B
58.	Priponești, GL	Grivița, IV, 13B	46.082222	27.491111	50/-	50 Pl 50 Fe	12	E, E+P, L	2 PNw + 1 B
59.	Bălcuța, BC	Sascut, I, 14A	46.185833	27.036944	360/N	100 Pa	35	L	1 PNw
		Sascut, I, 14B	46.185669	27.035126	380/NE	70 Fs 30 Qp	55	E, E+P	1PNw + 1 B
60.	Ștei, HD	Retezat, III, 48	45.566033	22.737778	490/W	30 Fs 30 Qc 20 Qp 20 Cb	80	L	1 PNw
		Retezat, III, 50A	45.563611	22.735833	460/W	30 Qp 30 Pa 20 Fs 20 Cb	25	E	1 B
		Retezat, III, 100B	45.563714	22.730278	505/S	90 Pa 10 Qc	40	E+P	1 PNw
		Retezat, III, 109C	45.563889	22.732778	460/-	Administrative area	-	D, T	2 PNw
61.	Turnu Ruieni, CS	INCDS, IV, 24A	45.411944	22.344722	510/NW	100 Fs	100	D, T	2 PNw
		INCDS, IV, 26A	45.407222	22.344444	430/SE	40 Fs 30 Pn 20 Pa 10 Aps	30	E+P, L	2 PNw
		INCDS, IV, forested pasture	45.405833	22.348611	370/W	20 Cb 20 Fs 20 Qc 10 Sc 10 Tc 10 Bp 10 Ai	20	E	1 B
62.	Feneș_1, CS	Fruit orchards	45.187222	22.338889	450/NW	Mp, Pc, Pav, Pd	20	E, E+P	1 PNw + 1 B
		Community forest	45.185278	22.340000	490/N	80 Fs 10 Qc 10 Bp	70	E	1 B
63.	Feneș_2, CS	Adsilva wood processing factory	45.192778	22.348889	385/-	WPF soft- and hardwood	-	D, E+P, T	1 PNw + 2 B
64.	Caransebeș, CS	Massiv wood processing factory	45.377778	22.241111	220/-	WPF only hardwood	-	E, E+P, L	2 PNw + 1 B
65.	Valea Minișului, CS	Anina, III, 80	45.025278	21.895556	475/SW	60 Fs 30 Cb 10Aps	20	E	1 B
		Anina, III, 83A	45.023056	21.898333	500/SW	60 Fs 20 Tc 10 Cb 10 Dt	105	D, T	2 PNw
		Anina, III, 83B	45.022222	21.898889	475/S	30 Pa 30 Pn 20 Ps 10 Ptx 10 Cb	40	E+P	1 PNw
		Anina, III, 83E	45.022500	21.895556	485/S	90 Fs 10 Cb	50	L	1 PNw
66.	Drobeta Turnu-Severin, MH	Wood Yard	44.609722	22.733333	70/-	WY oak, beech, ash	-	E, E+P, L	2 PNw + 1 B
**Traps installed in 2017**									
67.	Dumbrăveni, SB	Dumbrăveni, IV, 85B	46.250556	24.564722	415/SE	50 Ps 30 Qp 20 Cb	105	All 5	5 PNw
68.	Târnăveni, MS	Luduș, I, 65D	46.371389	24.265556	370/W	80 Fe 10 Pn 10 Dt	45	All 5	5 PNw
69.	Praid, HR	Praid, I, 257A	46.554444	25.120556	520/SE	50 Ps 20 Qp 10 Fe 10 Ac 10 Dt	100	All 5	5 PNw
70.	Vidra, VN	INCDS, I, 124A, B	46.900556	26.980278	340/E	90 Qp 10 Tc	70	All 5	5 PNw
71.	Ineu, AR	Ineu, Castel Forest	46.385556	21.875833	140/-	40 Qc 30 Qf 30 Qr	65-100	E, E+P, L	3 PNw
		Ineu, Castel Forest	46.385556	21.875556	140/-	50 Pa 30 Qc 20 Qr	40-75	D, T	2 PNw
72.	Tinca, BH	Tinca, I, 97B	46.809722	21.848333	135/-	100 Qc	70	All 5	5 PNw
73.	Marghita, BH	Marghita, I, 32 B	47.364167	22.334444	170/-	30 Qc 30 Qp 30 Qr 10 Cb	40	All 5	5 PNw
74.	Băile Felix, BH	Oradea, V, 25 C	46.993889	21.974444	170/-	70 Ps 30 Qru	100	All 5	5 PNw
75.	Babadag, TL	INCDS, Tulcea Experimental Basis, 57A	44.845000	28.704167	130/W	40 Qpf 30 Qpb 20 Fo 10 Dt	90	All 5	5 PNw
76.	Andrășești, IL	Slobozia, I, 77A	44.573947	27.161889	30/-	90 Qr 10 Dt	80	All 5	5 PNw
77.	Bărăgan, CL	INCDS, Bărăgan Experi-mental Basis, II, 6B	44.433122	27590982	50/-	30 Qpf 30 Fe 10 Fo 30 Dt	60	D, E, E+P, T	4 PNw

78.	Mădărş, SM	Satu Mare, II, 79F	47.709640	22.832671	125/-	100 Qr	50	E, E+P	2 PNw
		Satu Mare, II, 80E	47.709835	22.832617	125/-	50 Qr 30 Fe 20 Cb	20	D, L, T	3 PNw
79.	Turț, SM	Livada, II, 69A	47.973682	23.256832	490/E	100 Fs	75	E, E+P	2 PNw
		Livada, II, 69B	47.970326	23.255600	520/SE	100 Pa	45	D, L, T	3 PNw
80.	Cavnic, MM	Baia Sprie, III, 92A	47.665138	23.884747	950/S	100 Fs	80	All 5	5 PNw
81.	Fântânele, BN	INCDS, Lechința Experi-mental Basis, V, 30D	46.947441	24.243394	500/N	40 Pa 40 Qp 20 Cb	35	All 5	5 PNw
82.	Valea Ierii, CJ	Valea Ierii, IV, 12B	46.602910	23.283269	850/NW	50 Fs 40 Pa 10 Bp	25	All 5	5 PNw
**Other our data**									
83.	Groşii Noi, AR	Bârzava, IV, 98-100 (2009)	46.169844	22.103121	520/S	50 Qp 50 Fs	140	-	20 PNpw
84.	Voievodeasa, SV	Marginea, II, 5A (2011)	47.821447	25.688694	850/SE	90 Fs 10 Aa	130	-	20 PNpw
		Marginea, II, 5A (2012)	47.821447	25.688694	850/SE	90 Fs 10 Aa	130	-	20 PNpw
		Marginea, II, 5A (2018)	47.821447	25.688694	850/SE	90 Fs 10 Aa	130	E	9 PNpw
		Marginea, II, 5C (2016)	47.815068	25.697613	625/SE	50 Pa 50 Aa	60	E	9 PNw
85.	Palma, SV	Marginea, II, 61 B (2018)	47.760090	25.637700	880/NW	70 Fs 20 Pa 1 Aa	75	E	9 B
86.	Ciumârna, SV	Vama, III, 355A (2015)	47.694369	25.587906	825/SE	100 Pa	115	Tetropium	20 PNw
87.	Demacuşa, SV	Tomnatic, I, 90B (2015)	47.704722	25.408333	870/SE	100 Pa	100	T	5 PNw + 5 T
88.	Bobeica, SV	Cârlibaba, VII, 49I (2014)	47.713219	25.076333	1200/S	100 Pa	115	E, L	6 T
		Cârlibaba, VII, 75C (2017)	47.675925	25.123916	1195/N	100 Pa	95	T	15 Dif
89.	Cârlibaba, SV	Cârlibaba, VI, 133A (2017)	47.600453	25.191978	1270/NE	100 Pa	95	L	14 PNw
90.	Iacobeni, SV	Iacobeni, VI, 5A (2016)	47.411472	25.311861	985/W	100 Pa	100	T, T+E+P	20 PNw
91.	Cacica, SV	Solca, II, 9B (2014)	47.632289	25.920764	460/E	40 Aa 40 Pa 20 Fs	125	E	3 T
		Solca, II, 9B (2015)	47.632289	25.920764	460/E	40 Aa 40 Pa 20 Fs	125	L	3T
		Solca, I, 56A (2015)	47.717784	25.839566	505/NE	60 Pa 30 Aa 10 Dt	95	D, T, D+T	15 PNw
		Solca, II, 9A (2017)	47.615031	25.918939	480/N	90 Fs 10 Aa	115	E	5 PNw + 5 B
		Solca, II, 9A (2018)	47.609669	25.914521	520/NW	90 Fs 10 Aa	115	E	9 B
		Solca, II, 2D (2018)	47.589439	25.927397	620/NW	80 Fs 10 Aa 10 Pa	65	E	9B
		Solca, II, 10 (2013)	47.616550	25.916935	426/NE	100 Pa	30	D	5 PNw
92.	Căpățâneni, AG	Vidraru, VI, 42B, 43 B (2015)	45.355678	24.687756	1520/S	100 Pa	125	L	3 PNw
93.	Roznov, NT	Roznov, II, 162A (2017)	46.806061	26.372188	410/N	80 Fs 20Aa	110	E	9 PNw
94.	Valea Budului, BC	Fântânele, I, 55C (2018)	46.595000	26.799444	265/S	80 Qp 10 Tc 10 Cb	120	E+L	4 PNw
95.	Mihăeşti, AG	INCDS, XII, 143C (2018)	44.954092	24.967584	440/-	80 Ps 20 Qp	35	L	3 PNw
96.	Braşov, BV	Warthe Hill (2018)	45.645000	25.569444	685/NW	60 Qp 40 Fs	90	L	4 PNw
97.	Todireşti, SV	Pătrăuți, II, 28 (2013)	47.714520	26.033568	406/-	100 Pa	45	D	5 PNw
98.	Solca, SV	Solca, I, 39 C (2013)	47.699889	25.795933	602/S	100 Pa	85	D	5 PNw
99.	Sucevița, SV	Marginea, III, 194B (2013)	47.777919	25.714744	614/NW	100 Pa	80	D	5 PNw
		Marginea, II, 53 (2013)	47.772863	25.634523	807/SE	100 Pa	60	D	5 PNw
		Marginea, II, 70 (2013)	47.743168	25.644788	1002/N	80 Pa 20 Fs	90	D	5 PNw
100.	Ionu, SV	Vama, II, 91 D (2013)	47.633385	25.497985	832/NE	100 Pa	80	D	5 PNw
		Vama, II, 84 C (2013)	47.608227	25.474411	1032/SW	100 Pa	100	D	5 PNw

		Vama. II, 82I (2013)	47.617857	25.481843	1168/SE	100 Pa	100	D	5 PNw
		Vama, II, 87A (2013)	47.628156	25.483587	1207/S	100 Pa	110	D	5 PNw
101.	Fetești, SV	Adâncata, VI, 34 (2011)	47.725854	26.324818	400/-	100 Pa	40	ID EXP	30 PNw
		Adâncata, VI, 34 (2012)	47.725854	26.324818	400/-	100 Pa	40	ID EXP	30 PNw
102.	Calafindești, SV	Pătrăuți, III, 22 (2011)	47.848087	26.143896	490/-	100 Pa	40	ID EXP	30 PNw
		Pătrăuți, III, 22 (2012)	47.848087	26.143896	490/-	100 Pa	40	ID EXP	30 PNw
103.	Zamostea, SV	Adâncata, VIII, 4 (2011)	47.879464	26.142520	375/-	100 Pa	40	ID EXP	30 PNw
104.	Mitocaș, SV	Adâncata, VII, 5 (2012)	47.750017	26.253819	440/-	100 Pa	35	ID EXP	30 PNw
105	Salcea, SV	Pepiniera Salcea (2017)	47.626986	26.394888	286/-	Conifers and broadleaves	0-10	D, E, E+P, L	9 PNw
106.	Nechit, NT	Tazlău, I, 96 (2017)	46.764826	26.342328	632/NE	80 Pa 10 Aa 10 Fs	85	T	5 PNw + 5 T
107.	Breaza, SV	Breaza, III, 110B (2016)	47.659471	25.332193	1070/W	90 Pa 10 Ps	125	E+P	10 PNw
108.	Barnar, SV	Crucea, III, 159A (2016)	47.272175	25.481011	1100/S	100 Pa	90	E+P	10 PNw
109.	Argel, SV	Moldovița, II, 234C (2016)	47.786913	25.436098	950/SW	60 Pa 30 Aa 10 Fs	100	E+P	10 PNw
110.	Solonețu Nou, SV	Solca, II, 25H (2016)	47.653241	25.856584	466/N	100 Pa	30	D	3 PNw
111.	Pângărați, NT	Logs yard (2016)	46.919079	26.289511	365/-	-	-	T, D, E, E+AP	9 PNw
112.	Vileacu de Beiuș, BH	Fruit tree orchard (2018)	46.678506	22.224436	145/-	Mp, Pc, Pp	5	-	-

County: AB – Alba, AG – Argeș, AR – Arad, BC – Bacău, BH – Bihor, BN – Bistrița-Năsăud, BT – Botoșani, BV – Brașov, BZ – Buzău, CJ – Cluj, CL – Călărași, CS – Caraș-Severin, CT – Constanța, CV – Covasna, DB – Dâmbovița, DJ – Dolj, GJ – Gorj, GL – Galați, GR – Giurgiu, HD – Hunedoara, HR – Harghita, IL – Ilfov, IS – Iași, MH – Mehedinți, MM – Maramureș, MS – Mureș, NT – Neamț, PH – Prahova, SB – Sibiu, SM – Satu-Mare, SV – Suceava, TL – Tulcea, TM – Timiș, VL – Vâlcea, VN – Vrancea, VS – Vaslui.

Forest and fruit tree species: Aa - *Abies alba* Mill., Ac – *Acer campestre* L., Ag – *Alnus glutinosa* (L.) Gaertn., Ai – *Alnus incana* (L.) Moench, An – *Acer negundo* L., Ap – *Acer platanoides* L., Aps - *Acer pseudoplatanus* L., Bp – *Betula pendula* Roth, Cb – *Carpinus betulus* L., Fe – *Fraxinus excelsior* L., Fo – *Fraxinus ornus* L., Fs - *Fagus sylvatica* L., Jr – *Juglans regia* L., Ld - *Larix decidua* Mill., Mp – *Malus pumila* Mill., Pa - *Picea abies* (L.) H. Karst., Pav – *Prunus avium* (L.) L., Pc – *Pyrus communis* L., Pd – *Prunus domestica* L., Pl – *Plopus* sp., Pm – *Pseudotsuga menziesii* (Mirb.) Franco, Pn – *Pinus nigra* J.F. Arnold, Pp – *Prunus persica* (L.) Batsch, Ps – *Pinus sylvestris* L., Pst – *Pinus strobus* L., Pt – *Populus tremula* L., Qc – *Quercus cerris* L., Qf – *Quercus fraineto* Ten., Qp – *Qercus petraea* (Matt.) Liebl., Qpb – *Quercus pubescens* Willd., Qpf – *Quercus robur* subsp. *Pedunculiflora* (K. Koch) Menitsky, Qr – *Quercus robur* L., Qru – *Quercus rubra* L., Rp – *Robinia pseudoacacia* L., Sc – *Salix caprea* L., Tc – *Tilia cordata* Mill., Tt – *Tilia tomentosa* Moench, Dr- Other conifers; Dt - Other deciduos trees; OPS – Ornamental plant store; WPF – wood processing factory; WY – wood yard

Lure types: D – AtraDUP, E – Ethanol, E+P – Ethanol + Alpha-Pinene, L – AtraLINEA, T – AtraTYP, Tetropium – Fuscumol, Fuscumol + Alpha-Pinene + Ethanol, Ethanol + Apha-Pinene, ID EXP. – Experimental lures for *I. duplicatus* (diverse combinations of Ipsdienol, E-Myrcenol, Methyl-Butenol, Alpha-Pinene and Limonene) (Duduman, 2014[1]).

Trap types: PNd – dry Panel trap, PNw – wet Panel trap, B – Bottle trap, PNpw – wet Panel traps of polyethylene film, T – Theysohn, 15 Dif – 5 PNw + 5 MultiWit + 5 Lindren multiple funnel traps.

[1] Duduman, M.-L. 2014. Field response of the northern spruce bark beetle *Ips duplicatus* (Sahlberg) (Coleoptera: Curculionidae, Scolytinae) to different combinations of synthetic pheromone with (−)-α-pinene and (+)-limonene. Agricultural and Forest Entomology, 102-109 [191].

Table S2. Locations of *Ips duplicatus* major foci between 2005 and 2014

No.	Location, county	Forest district, production unit	Coordinates (º)		Elevation (m)
			N	E	
F1.	Solca, SV	Solca, I Solca	47.7029	25.8157	500-700
F2.	Cacica, SV	Solca, II Cacica	47.6175	25.9273	400-600
F3.	Ilişeşti, SV	Solca, III Ilişeşti	47.6263	26.0345	350-500
F4.	Putna, SV	Putna, I Putna	47.8186	25.6268	400-800
		Putna, II Putnisoara	47.8266	25.6038	400-800
F5.	Todireşti, SV	Patrauti, II Todiresti	47.7195	26.0388	400-450
F6.	Calafindeşti, SV	Patrauti, III Darmanesti	47.7471	26.1529	400-500
F7.	Feteşti, SV	Adancata, VI Adâncata	47.7150	26.3050	300-350
F8.	Zvoriştea, SV	Adancata, VII Zvoriştea	47.8048	26.2223	350-450
F9.	Zamostea, SV	Adancata, VIII Zamostea	47.8358	26.1784	400-500
F10.	Probota, SV	Dolhasca, I Probota	47.3635	26.6100	300-350
F11.	Dolheşti, SV	Dolhasca, II Dolhesti	47.4217	26.5496	300-400
F12.	Liteni, SV	Dolhasca, III Liteni	47.5103	26.4834	280-350
F13.	Sireţel, IS	Dolhasca, IV Siretel	47.4007	26.7367	300-400
F14.	Dolhasca, SV	Dolhasca, V Dolhasca	47.3982	26.6380	350-450
F15.	Preuteşti, SV	Falticeni, V Falticeni	47.4412	26.3591	300-400
F16.	Râşca, SV	Falticeni, VII Slatioara	47.3828	26.1470	450-550
		Râsca, II Moişa	47.3194	26.2449	450-650
F17.	Mălini, SV	Malini, I Mălini	47.4523	25.9795	450-700
F18.	Dumbrăveni, SV	Râsca, III Râşcuţa	47.3252	26.1144	400-700
F19.	Buda, SV	Râsca, V Râşca Mare	47.3637	26.1327	450-650
F20.	Negrileasa, SV	Stulpicani, II Negrileasa	47.3483	25.8602	750-900
F21.	Slatina, SV	Gura Humorului, I Capul Câmpului	47.4558	25.9928	500-700
F22.	Voroneţ, SV	Gura Humorului, II Voroneţ	47.4986	25.8834	550-800
F23.	Mănăstirea Humorului, SV	Gura Humorului, III Humor	47.6240	25.8611	550-800
F24.	Păltinoasa, SV	Gura Humorului, V Paltinoasa	47.5535	25.9646	500-650
F25.	Frasin, SV	Frasin, III Sălătruc	47.5397	25.7765	550-800
F26.	Tătăruşi, IS	Pascani, II Tătăruşi	47.1426	26.2594	350-400
F27.	Todireşti, IS	Pascani, III Hărmăneşti	47.3390	26.8195	350-400
F28.	Târgu Neamţ, NT	Neamţ, IV Cetatea Neamţului	47.2247	26.3528	400-550
F29.	Văratec, NT	Bisericesc, Neamţ, III Agapia-Bistriţa	47.1426	26.2594	600-800
F30.	Stăniţa, NT	Roman, V Vulpăneşti	46.9461	27.1623	200-300
F31.	Gâceşti, VS	Băceşti, I Gârceni	46.7404	27.3111	240-280
F32.	Oniceni, NT	Băceşti, V Oniceni	46.7877	27.148	200-300
F33.	Poienari, NT	Băceşti, VI Poienari	46.8794	27.1053	340-360
F34.	Ţibăneşti, VS	Băceşti, VII Ţibăneşti	46.9470	27.2581	300-380
F35.	Chiţoc, VS	Brodoc, I Chiţoc	46.5769	27.6946	150-250
F36.	Floreşti, VS	Vaslui, IV Floreşti	46.5363	27.5509	400-420
F37.	Ivăneşti, VS	Vaslui, V Ivăneşti	46.6089	27.4881	270-400
F38.	Bonţeşti, VN	Focşani, VI Coteşti	45.6871	27.0086	500-600
F39.	Câmpuri, VN	Soveja, I Câmpuri	45.9953	26.7171	600-850
F40.	Tomeşti, IS	Ciurea , I Tomeşti	47.1190	27.6674	150-250
F41.	Poieni, IS	Ciurea, II Poieni	47.0664	27.7177	250-280
F42.	Pocreaca, IS	Dobrovăţ, I Nastea	46.9344	27.7353	200-250
F43.	Dobrovăţ, IS	Dobrovăţ, II Pietrosu	46.9856	27.6574	200-300
F44.	Zebreni, IS	Hârlău, I Cotnari	47.3751	26.8324	280-330
F45.	Hârlău, IS	Hârlău, II Maxut	47.4133	26.8713	220-300
F46.	Humosu, IS	Hârlău, III Humosu	47.4447	26.7201	320-380
F47.	Poiana Deleni, IS	Hârlău, IV Deleni	47.4801	26.8243	270-290
F48.	Moţca, IS	Paşcani, I Paşcani	47.2649	26.6217	310-330
F49.	Iorcani, IS	Paşcani, II Tătăruşi	47.3609	26.5342	410-440
F50.	Criveşti, IS	Paşcani, III Hărmăneşti	47.3012	26.7778	280-380

F51.	Todirel, IS	Padureni, IV Barnova	47.0465	27.6419	230-250
F52.	Fărcășești, IS	Podu Iloaiei, I Strunga	47.1731	26.9149	270-290
F53.	Brăești, IS	Podu Iloaiei, II Brăești	47.1150	27.1105	180-200
F54.	Popești, IS	Podu Iloaiei, III Popești	47.1023	27.2853	160-170
F55.	Mădârjac, IS	Podu Iloaiei - U,P, V Gheorghițoaia	47.0449	27.2376	210-230
F56.	Răducăneni, IS	Răducăneni - IV Bunești	46.9387	27.9004	320-330
F57.	Poiana, BT	Dorohoi, I Văculești	47.8544	26.4037	270-280
F58.	Copălău, BT	Botoșani, II Coșula	47.6017	26.8277	180-200
F59.	Oneaga, BT	Botoșani, III Cristești	47.5738	26.7067	260-320
F60.	Tudora, BT	Mihai Eminescu, I Tudora	47.4832	26.6733	350-390
F61.	Serafinești, BT	Mihai Eminescu, II Vorona	47.6448	26.6504	140-170
F62.	Daneş, MS	Sighișoara, V Sighișoara	46.2069	24.7390	470-490

County: BT – Botoșani. BV – Brașov. IS – Iași. MS – Mureş. NT – Neamț. SV – Suceava. VN - Vrancea. VS – Vaslui.

Table S3. Total captures of *I. duplicatus*, *X. germanus* and *N. acuminatus*

No.	Location	Number of traps	Monitored period	Number of ... beetles		
				I. duplicatus	*X. germanus*	*N. acuminatus*
Traps set up in 2015						
1.	Almaș	3	20.06-26.07.15	0	470	0
2.	Roman	3	20.06-26.07.15	0	0	0
3.	Sânsimion	1	21.06-19.07.15	0	0	0
		2	21.06-19.07.15	0	0	0
4.	Măgura	3	30.06-15.08.15	0	0	0
5.	Pojoga	3	23.06-16.07.15	0	0	0
6.	Tamași	3	13.06-11.07.15	0	0	0
7.	Porumbacu de Sus_1	3	23.06-20.07.15	0	0	0
8.	Glâmboaca	3	18.06-16.07.15	0	0	0
9.	Haleș	3	01.07-28.07.15	0	0	0
10.	Negrița	3	11.06-10.07.15	0	2	0
11.	Bădeanca	3	11.06-10.07.15	0	1	0
12.	Warthe	3	29.06-15.07.15	0	262	0
13.	Bunloc	3	11.06-30.06.15	0	100	0
Traps set up in 2016						
14.	Porumbacu de Sus_2	5	12.05-28.09.16	508	46	0
15.	Brașov_1	5	11.05-12.10.16	265	1,058	0
16.	Brașov_2	5	11.05-23.09.16	4,551	29	0
17.	Zăbrătău	5	19.05-29.09.16	2	442	0
18.	Reci	5	24.05-12.10.16	1,357	1	0
19.	Reghin	5	18.05-22.09.16	537	0	0
20.	Lunca Bradului	5	18.05-22.09.16	899	2	0
21.	Borsec	5	18.05-21.09.16	2,145	0	0
22.	Zăval	3	10.05-07.10.16	0	0	0
23.	Balasan	3	11.05-05.10.16	0	0	1
24.	Arginești	5	13.05-30.09.16	0	0	0
25.	Tismana	5	17.05-03.10.16	0	131	0
26.	Crivina	3	18.05-10.10.16	0	0	1
27.	Băbeni	5	19.05-06.10.16	2	1	0
28.	Firești	5	19.05-06.10.16	0	2	0
29.	Craiova_1	3	20.05-29.09.16	0	0	0
30.	Craiova_2	4	20.05-29.09.16	0	0	0
31.	Borș	5	19.05-10.09.16	0	0	0
32.	Cluj-Napoca	5	31.05-20.09.16	0	1	0
33.	Sebeș	5	11.05-20.09.16	2,787	0	0
34.	Ștefănești	3	16.05-22.09.16	0	21	0
35.	Mihăești	5	18.05-20.09.16	0	1	0
36.	Giurgiu	3	18.05-20.09.16	0	1	0
37.	Găești	3	18.05-20.09.16	0	1	0
38.	Agigea	5	24.05-21.09.16	9	0	0
39.	Călărași	3	24.05-21.09.16	0	0	0
40.	Oltenița	5	24.05-21.09.16	0	0	10
41.	Băicoi	5	25.05-22.09.16	85	12	0
42.	Otopeni	5	30.05-22.09.16	3	0	0
43.	Văliug	5	12.05-30.09.16	1	24	0
44.	Timișoara	5	19.05-27.09.16	2	0	0
45.	Cenad	4	07.06-26.09.16	0	0	0
46.	Curtici	4	07.06-28.09.16	0	0	1
47.	Ilia	4	08.06-29.09.16	3	3	0
48.	Oneaga	3	05.05-22.09.16	0	18	0

49.	Iași	5	09.05-22.09.16	19	0	0
50.	Valea Ursului	3	09.05-22.09.16	0	0	0
51.	Sărata	5	10.05-28.09.16	328	422	0
52.	Hemeiuș	5	10.05-28.09.16	3,847	21	0
53.	Vermești	5	11.05-29.09.16	1,213	2	0
54.	Dumbrava Roșie	5	12.05-29.09.16	8,395	1	0
55.	Piatra Șoimului	3	12.05-29.09.16	7,041	10	0
56.	Crasna	3	16.05-20.09.16	0	0	0
57.	Bașta	3	16.05-22.09.16	0	0	0
58.	Priponești	3	17.05-20.09.16	1	0	0
59.	Bălcuța	5	18.05-28.09.16	329	5	0
60.	Ștei	5	20.05-16.09.16	0	0	0
61.	Turnu Ruieni	5	20.05-16.09.16	0	0	0
62.	Feneș_1	3	28.05-09.09.16	0	0	0
63.	Feneș_2	3	08.06-09.09.16	0	0	0
64.	Caransebeș	3	13.05-09.09.16	0	0	1
65.	Valea Minișului	5	10.06-08.09.16	0	671	0
66.	Drobeta Turnu-Severin	3	08.06-08.09.16	0	0	0
Traps set up in 2017						
67.	Dumbrăveni	5	04.04-11.09.17	5	0	0
68.	Târnăveni	5	04.04-14.09.17	18	0	0
69.	Praid	5	05.04-13.09.17	781	6	0
70.	Vidra	5	28.03-30.08.17	17	5	0
71.	Ineu	5	27.04-02.10.17	3	1	0
72.	Tinca	5	13.04-18.08.17	1	0	1
73.	Marghita	5	24.04-17.08.17	0	0	0
74.	Băile Felix	5	25.04-17.08.17	0	0	0
75.	Babadag	5	12.04-20.09.17	0	0	0
76.	Andrășești	5	12.04-19.09.17	0	0	0
77.	Bărăgan	4	13.04-19.09.17	0	0	0
78.	Mădăraș	5	04.04-10.08.17	0	0	0
79.	Turț	5	05.04-10.08.17	0	9	0
80.	Cavnic	5	06.04-10.08.17	0	0	0
81.	Fântânele	5	03.04-10.08.17	2	0	0
82.	Valea Ierii	5	30.05-28.07.17	0	0	0
Insect specimens collected in other own studies						
83.	Groșii Noi	20	06.05-20.05.09	0	14	0
84.	Voievodeasa	20	10.05-26.09.11	0	71	0
		20	03.05-20.09.12	0	97	0
		9	04.04-17.09.18	0	13,829	0
		9	19.05-28.06.16	0	511	0
85.	Palma	9	05.04-17.09.18	0	16,796	0
86.	Ciumârna	20	06.05-20.05.15	221	0	0
87.	Demacușa	10	04.05-15.06.15	5,749	0	0
88.	Bobeica	6	29.04-16.09.14	10	0	0
		15	02.05-26.06.17	0	1	0
89.	Cârlibaba	14	02.05-26.06.17	0	1	0
90.	Iacobeni	20	13.04.16.06.16	5	0	0
91.	Cacica	3	20.04-25.09.14	8	3,377	0
		3	03.04-18.05.15	12	15	0
		15	15.07-24.07.15	721	0	0
		10	16.05-22.06.17	0	23,329	0
		9	03.04-18.09.18	0	21,465	0
		9	04.04-18.09.18	0	66,836	0

		5	17.04-18.10.13	34437	0	0
92.	Căpățâneni	3	03.05-14.06.15	0	1	0
93.	Roznov	9	25.03-23.09.17	0	524	0
94.	Valea Budului	4	08.03-09.05.18	0	144	0
95.	Mihăiești	3	08.03-09.05.18	0	27	0
96.	Braşov	4	12.03-10.05.18	0	88	0
97.	Todirești	5	17.04-18.10.13	78140	0	0
98.	Solca	5	17.04-18.10.13	26500	0	0
99.	Sucevița	5<br>5<br>5	17.04-18.10.13<br>17.04-18.10.13<br>17.04-18.10.13	33240<br>18521<br>5420	0<br>0<br>0	0<br>0<br>0
100.	Ionu	5<br>5<br>5<br>5	20.04-03.10.13<br>20.04-03.10.13<br>20.04-03.10.13<br>20.04-03.10.13	7820<br>2180<br>220<br>178	0<br>0<br>0<br>0	0<br>0<br>0<br>0
101.	Fetești	30<br>30	16.05-28.07.11<br>19.05-19.06.12	17518<br>14999	0<br>0	0<br>0
102.	Calafindești	30<br>30	16.05-28.07.11<br>19.05-19.06.12	34529<br>17961	0<br>0	0<br>0
103.	Zamostea	30	16.05-28.07.11	36447	0	0
104.	Mitocaş	30	19.05-19.06.12	22737	0	0
105.	Salcea	6	06.03-11.09.17	3921	29	0
106.	Nechit	10	20.05.-01.07.17	639	0	0
107.	Breaza	10	29.04-04.10.16	171	0	0
108.	Barnar	10	28.04-04.10.16	5	0	0
109.	Argel	10	29.04-04.10.16	14	0	0
110.	Solonețu Nou	3	21.04-09.10.16	26118	0	0
111.	Pângărați	9	20.04-19.09.16	2342	0	0
112.	Vileacu de Beiuş	-	2018	-	3	-

Table S4. Climatic data of study locations for the years 1950-2017

No.	Location, county	1950-2017							1950-1983			1984-2017		
		MAT (°C)	MAP (mm)	I_{dM}	$T_{min-Jan}$	Summer (JJA)			MAT (°C)	MAP (mm)	I_{dM}	MAT (°C)	MAP (mm)	I_{dM}
						T_{mean-S}	T_{max-S}	MAP-S						
1	Almaş, NT	7.9	536.8	30.0	-8.1	18.3	24.6	222.6	7.8	529.9	29.8	8.0	543.6	30.2
2	Roman, NT	9.1	521.6	27.3	-6.8	19.8	26.1	201.9	8.9	502.9	26.6	9.3	540.2	28.0
3	Sânsimion, HR	5.9	561.4	35.3	-10.5	16.0	22.4	228.0	6.1	575.1	35.6	5.6	547.8	35.0
4	Măgura, BZ	9.2	526.1	27.3	-6.9	19.9	25.9	194.0	9.4	529.6	27.3	9.1	522.6	27.3
5	Pojoga, HD	9.8	594.0	30.0	-5.1	19.5	26.0	202.0	9.8	588.0	29.7	9.9	600.0	30.2
6	Tamaşi, BC	9.2	523.1	27.2	-6.7	19.9	26.2	201.2	9.1	495.8	26.0	9.4	550.3	28.4
7	Porumbacu de Sus_1, SB	7.3	647.0	37.4	-8.4	17.0	22.7	245.0	7.4	664.7	38.2	7.2	629.4	36.6
8	Glâmboaca, SB	8.9	572.7	30.2	-7.7	19.0	25.3	224.7	9.0	572.8	30.1	8.8	572.6	30.4
9	Haleş, BZ	10.6	516.4	25.1	-5.8	21.4	27.4	186.1	10.7	518.1	25.0	10.4	514.7	25.2
10	Negriţa, DB	4.2	692.2	48.9	-10.4	13.6	18.6	263.3	4.3	738.5	51.7	4.0	646.0	46.1
11	Bădeanca, AG	4.2	692.2	48.9	-10.4	13.6	18.6	263.3	4.3	738.5	51.7	4.0	646.0	46.1
12	Warthe, BV	6.9	591.8	35.0	-9.2	17.1	23.1	228.7	7.1	606.2	35.4	6.7	577.3	34.6
13	Bunloc, BV	6.9	591.8	35.0	-9.2	17.1	23.1	228.7	7.1	606.2	35.4	6.7	577.3	34.6
14	Porumbacu de Sus_2, SB	7.3	647.0	37.4	-8.4	17.0	22.7	245.0	7.4	664.7	38.2	7.2	629.4	36.6
15	Braşov_1, BV	6.9	591.8	35.0	-9.2	17.1	23.1	228.7	7.1	606.2	35.4	6.7	577.3	34.6
16	Braşov_2, BV	6.9	591.8	35.0	-9.2	17.1	23.1	228.7	7.1	606.2	35.4	6.7	577.3	34.6
17	Zăbrătău, CV	6.2	586.8	36.2	-9.5	16.3	22.2	226.9	6.4	606.5	37.0	6.0	567.0	35.4
18	Reci, CV	7.0	551.9	32.4	-9.5	17.4	23.8	218.9	7.3	558.8	32.3	6.8	545.0	32.4
19	Reghin, MS	8.2	546.8	30.1	-8.4	18.2	24.7	217.3	8.2	537.1	29.5	8.2	556.4	30.6
20	Lunca Bradului, MS	5.3	610.5	40.0	-10.2	14.9	20.9	252.7	5.3	636.8	41.6	5.2	584.2	38.3
21	Borsec, HR	4.5	606.3	41.8	-10.6	14.2	19.9	254.4	4.5	638.7	43.9	4.5	573.9	39.6
22	Zăval, DJ	11.8	515.5	23.6	-4.7	22.7	29.4	140.5	11.6	515.8	23.9	12.0	515.1	23.4
23	Balasan, DJ	11.7	551.3	25.4	-4.4	22.5	29.0	139.9	11.5	553.9	25.8	11.9	548.7	25.0
24	Argineşti, MH	12.0	619.8	28.2	-4.7	22.7	28.4	166.8	12.1	639.5	28.9	11.8	600.1	27.5
25	Tismana, GJ	7.8	735.5	41.2	-7.0	17.5	22.4	236.2	7.9	766.2	42.7	7.7	704.7	39.7
26	Crivina, MH	11.8	616.6	28.2	-3.8	22.4	28.7	144.1	11.6	638.4	29.6	12.1	594.8	26.9
27	Băbeni, VL	10.9	637.3	30.5	-5.4	21.3	27.2	201.8	11.0	644.9	30.7	10.8	629.7	30.3
28	Fireşti, VL	9.8	662.1	33.5	-6.2	19.9	25.7	224.0	9.9	674.0	33.9	9.7	650.3	33.1
29	Craiova_1, DJ	11.4	571.2	26.6	-4.9	22.3	28.5	162.8	11.4	566.0	26.4	11.5	576.4	26.8
30	Craiova_2, DJ	11.4	571.2	26.6	-4.9	22.3	28.5	162.8	11.4	566.0	26.4	11.5	576.4	26.8
31	Borş, BH	10.3	502.0	24.7	-4.7	20.4	26.6	171.0	10.1	492.4	24.5	10.6	511.6	24.9

32	Cluj-Napoca, CJ	8.9	547.4	28.9	-6.8	18.8	25.2	214.6	8.8	522.8	27.8	9.0	572.0	30.1
33	Sebeș, AB	8.8	595.7	31.7	-7.2	18.6	24.6	223.4	8.9	601.1	31.9	8.7	590.3	31.6
34	Ștefănești, IF	11.2	556.7	26.2	-5.3	22.2	28.8	175.1	11.5	549.1	25.5	11.0	564.3	26.9
35	Mihăești, AG	10.2	595.7	29.5	-6.0	20.7	27.0	199.0	10.3	598.3	29.5	10.1	593.1	29.5
36	Giurgiu, GR	11.9	528.2	24.2	-4.5	22.6	28.9	152.9	11.8	525.4	24.1	11.9	531.0	24.3
37	Găești, DB	10.9	546.3	26.1	-5.6	21.8	28.3	174.8	11.1	547.0	25.9	10.7	545.6	26.3
38	Agigea, CT	12.0	400.2	18.2	-2.2	22.1	26.4	98.0	11.8	377.3	17.3	12.1	423.1	19.1
39	Călărași, CL	12.0	469.5	21.4	-4.0	22.7	28.9	136.3	12.1	452.6	20.5	11.9	486.4	22.3
40	Oltenița, CL	12.0	496.2	22.6	-4.3	22.8	29.2	143.6	12.1	475.3	21.5	11.9	517.2	23.7
41	Băicoi, PH	9.1	563.4	29.5	-7.2	19.6	25.9	201.3	9.3	569.6	29.5	8.9	557.2	29.5
42	Otopeni, IF	11.2	556.7	26.2	-5.3	22.2	28.8	175.1	11.5	549.1	25.5	11.0	564.3	26.9
43	Văliug, CS	7.7	730.6	41.2	-6.4	17.2	22.4	235.2	7.6	746.6	42.3	7.9	714.6	40.0
44	Timișoara, TM	10.9	547.4	26.2	-4.1	20.8	27.3	177.3	10.7	539.7	26.1	11.1	555.1	26.3
45	Cenad, TM	11.0	485.5	23.1	-4.0	21.0	27.4	157.1	10.7	473.5	22.9	11.3	497.4	23.3
46	Curtici, AR	10.8	492.9	23.7	-4.3	20.9	27.4	160.6	10.6	473.5	23.0	11.0	512.2	24.3
47	Ilia, HD	9.1	605.2	31.7	-5.9	18.7	25.0	210.2	9.1	605.5	31.8	9.1	604.9	31.7
48	Oneaga, BT	9.4	509.5	26.3	-6.4	20.0	26.2	206.6	9.1	498.5	26.1	9.7	520.6	26.4
49	Iași, IS	9.7	518.7	26.3	-6.1	20.6	26.9	195.1	9.4	514.4	26.5	10.0	523.0	26.2
50	Valea Ursului, IS	9.6	509.8	26.0	-6.3	20.4	26.7	196.5	9.3	499.8	25.9	9.8	519.8	26.2
51	Sărata, BC	9.5	515.3	26.5	-6.7	20.2	26.6	202.0	9.4	483.6	25.0	9.6	547.1	27.9
52	Hemeiuș, BC	9.5	515.3	26.5	-6.7	20.2	26.6	202.0	9.4	483.6	25.0	9.6	547.1	27.9
53	Vermești, BC	6.3	548.6	33.6	-9.8	16.6	22.9	221.3	6.5	551.3	33.5	6.2	546.0	33.7
54	Dumbrava Roșie, NT	7.9	530.4	29.6	-8.3	18.4	24.8	217.3	7.9	517.6	29.0	7.9	543.3	30.3
55	Piatra Șoimului, NT	7.9	530.4	29.6	-8.3	18.4	24.8	217.3	7.9	517.6	29.0	7.9	543.3	30.3
56	Crasna, VS	9.9	500.2	25.2	-5.8	20.8	26.9	175.9	9.6	485.5	24.7	10.1	514.9	25.6
57	Bașta, NT	9.2	509.9	26.6	-6.9	19.9	26.3	202.1	9.1	487.6	25.6	9.3	532.3	27.5
58	Priponești, GL	9.9	488.5	24.5	-6.1	20.8	27.0	177.5	9.8	466.3	23.5	10.1	510.7	25.4
59	Bălcuța, BC	10.0	487.0	24.4	-6.2	20.8	27.1	180.1	9.9	464.7	23.4	10.0	509.4	25.4
60	Ștei, HD	7.9	693.1	38.7	-6.7	17.4	23.0	239.1	7.9	702.7	39.3	7.9	683.6	38.2
61	Turnu Ruieni, CS	9.5	699.2	35.9	-5.2	19.0	24.9	230.6	9.4	701.2	36.1	9.5	697.2	35.8
62	Feneș_1, CS	8.6	714.6	38.4	-6.0	18.2	23.6	226.4	8.6	735.1	39.6	8.6	694.2	37.3
63	Feneș_2, CS	8.6	714.6	38.4	-6.0	18.2	23.6	226.4	8.6	735.1	39.6	8.6	694.2	37.3
64	Caransebeș, CS	9.5	695.9	35.7	-5.0	19.0	24.9	226.8	9.4	697.4	35.9	9.6	694.4	35.5
65	Valea Minișului, CS	9.0	699.3	36.8	-5.2	18.5	24.1	224.8	8.8	708.2	37.6	9.2	690.4	36.0
66	Drobeta Turnu-Severin, MH	11.7	644.6	29.6	-3.9	22.3	28.3	160.1	11.7	673.0	30.9	11.8	616.2	28.3
67	Dumbrăveni, SB	8.8	517.6	27.5	-8.1	19.0	25.7	208.1	8.9	506.3	26.7	8.7	528.9	28.3
68	Târnăveni, MS	9.1	520.4	27.3	-7.8	19.2	25.8	208.1	9.1	508.5	26.6	9.0	532.3	28.1
69	Praid, HR	5.9	571.7	36.0	-10.2	15.8	22.0	236.7	6.0	587.5	36.7	5.8	556.0	35.3
70	Vidra, VN	8.5	519.3	28.1	-7.6	19.1	25.3	196.2	8.5	513.5	27.7	8.4	525.2	28.5

71	Ineu, AR	10.6	506.8	24.6	-4.6	20.6	27.2	169.7	10.5	490.3	24.0	10.8	523.3	25.2
72	Tinca, BH	10.4	498.1	24.4	-4.7	20.4	26.8	169.3	10.2	481.2	23.8	10.6	515.0	25.0
73	Marghita, BH	10.2	543.8	27.0	-5.0	20.1	26.3	182.9	10.0	537.3	26.9	10.4	550.3	27.0
74	Băile Felix, BH	10.4	498.1	24.4	-4.7	20.4	26.8	169.3	10.2	481.2	23.8	10.6	515.0	25.0
75	Babadag, TL	11.1	416.8	19.7	-3.7	21.7	26.8	113.6	10.9	396.0	18.9	11.3	437.5	20.5
76	Andrăşeşti, IL	11.8	474.0	21.7	-4.5	22.8	28.9	148.1	12.0	461.7	21.0	11.7	486.3	22.4
77	Bărăgan, CL	12.0	445.5	20.2	-3.8	22.7	28.5	127.8	12.1	426.5	19.3	11.9	464.5	21.2
78	Mădărş, SM	10.4	641.7	31.5	-5.0	20.2	26.4	206.9	10.2	648.0	32.1	10.6	635.5	30.9
79	Turţ, SM	9.7	789.3	40.1	-5.5	19.4	25.5	250.2	9.5	842.2	43.2	9.9	736.3	37.0
80	Cavnic, MM	6.9	783.6	46.5	-7.5	16.2	21.9	286.4	6.6	815.8	49.1	7.1	751.5	43.9
81	Fântânele, BN	8.9	544.6	28.7	-7.3	18.9	25.4	211.7	8.9	524.1	27.7	9.0	565.1	29.8
82	Valea Ierii, CJ	6.7	635.3	38.0	-8.0	16.2	22.0	245.9	6.6	641.7	38.6	6.8	628.8	37.5
83	Groşii Noi, AR	9.5	572.2	29.3	-5.3	19.3	25.7	195.0	9.4	565.6	29.1	9.6	578.9	29.5
84	Voievodeasa, SV	6.7	647.4	38.9	-8.2	16.6	22.1	274.3	6.3	682.5	41.8	7.0	612.4	36.1
85	Palma, SV	6.7	647.4	38.9	-8.2	16.6	22.1	274.3	6.3	682.5	41.8	7.0	612.4	36.1
86	Ciumârna, SV	5.9	648.1	40.7	-8.9	15.7	21.3	275.5	5.7	686.2	43.8	6.1	610.0	37.8
87	Demacuşa, SV	4.8	713.4	48.2	-9.5	14.3	19.6	297.6	4.6	777.8	53.4	5.0	649.1	43.2
88	Bobeica, SV	4.0	769.1	55.0	-9.8	13.3	18.4	312.2	3.8	851.8	61.9	4.2	686.4	48.3
89	Cârlibaba, SV	4.0	769.1	55.0	-9.8	13.3	18.4	312.2	3.8	851.8	61.9	4.2	686.4	48.3
90	Iacobeni, SV	4.8	671.1	45.4	-9.7	14.3	19.8	281.4	4.6	721.6	49.4	5.0	620.5	41.4
91	Cacica, SV	7.2	593.8	34.6	-8.2	17.3	23.1	251.3	6.9	612.2	36.2	7.4	575.3	33.0
92	Căpăţâneni, AG	5.5	686.2	44.3	-9.4	15.0	20.2	255.7	5.6	720.2	46.2	5.4	652.1	42.4
93	Roznov, NT	7.9	530.4	29.6	-8.3	18.4	24.8	217.3	7.9	517.6	29.0	7.9	543.3	30.3
94	Valea Budului, BC	9.5	515.3	26.5	-6.7	20.2	26.6	202.0	9.4	483.6	25.0	9.6	547.1	27.9
95	Mihăeşti, AG	10.2	595.7	29.5	-6.0	20.7	27.0	199.0	10.3	598.3	29.5	10.1	593.1	29.5
96	Braşov, BV	6.9	591.8	35.0	-9.2	17.1	23.1	228.7	7.1	606.2	35.4	6.7	577.3	34.6
97	Todireşti, SV	8.1	555.6	30.7	-7.5	18.4	24.3	232.9	7.8	561.5	31.5	8.3	549.8	30.0
98	Solca, SV	7.2	593.8	34.6	-8.2	17.3	23.1	251.3	6.9	612.2	36.2	7.4	575.3	33.0
99	Suceviţa, SV	6.7	647.4	38.9	-8.2	16.6	22.1	274.3	6.3	682.5	41.8	7.0	612.4	36.1
100	Ionu, SV	4.8	713.4	48.2	-9.5	14.3	19.6	297.6	4.6	777.8	53.4	5.0	649.1	43.2
101	Feteşti, SV	8.5	539.7	29.1	-7.1	19.0	25.0	223.8	8.3	537.3	29.4	8.8	542.1	28.8
102	Calafindeşti, SV	8.2	572.0	31.4	-7.2	18.6	24.2	238.4	7.9	580.7	32.5	8.6	563.2	30.3
103	Zamostea, SV	8.2	572.0	31.4	-7.2	18.6	24.2	238.4	7.9	580.7	32.5	8.6	563.2	30.3
104	Mitocaş, SV	8.8	544.5	28.9	-6.8	19.3	25.2	224.4	8.5	542.8	29.4	9.2	546.2	28.5
105	Salcea, SV	8.5	539.7	29.1	-7.1	19.0	25.0	223.8	8.3	537.3	29.4	8.8	542.1	28.8
106	Nechit, NT	7.9	530.4	29.6	-8.3	18.4	24.8	217.3	7.9	517.6	29.0	7.9	543.3	30.3
107	Breaza, SV	4.8	713.4	48.2	-9.5	14.3	19.6	297.6	4.6	777.8	53.4	5.0	649.1	43.2
108	Barnar, SV	4.8	671.1	45.4	-9.7	14.3	19.8	281.4	4.6	721.6	49.4	4.9	620.5	41.5
109	Argel, SV	5.7	707.3	44.9	-8.7	15.5	20.8	299.4	5.4	763.4	49.4	6.1	651.3	40.6

110	Solonețu Nou, SV	7.2	593.8	34.6	-8.2	17.3	23.1	251.3	6.9	612.2	36.2	7.4	575.3	33.0
111	Pângărați, NT	7.9	530.4	29.6	-8.3	18.4	24.8	217.3	7.9	517.6	29.0	7.9	543.3	30.3
112	Vileacu de Beiuș, BH	9.3	543.7	28.2	-5.5	19.1	25.4	188.3	9.1	532.5	27.8	9.5	554.9	28.5
**Locations of *Ips duplicatus* major foci**														
1	Solca, SV	7.2	593.8	34.6	-8.2	17.3	23.1	251.3	6.9	612.2	36.2	7.4	575.3	33.0
2	Cacica, SV	7.2	593.8	34.6	-8.2	17.3	23.1	251.3	6.9	612.2	36.2	7.4	575.3	33.0
3	Ilișești, SV	8.1	555.6	30.7	-7.5	18.4	24.3	232.9	7.8	561.5	31.5	8.3	549.8	30.0
4	Putna, SV	6.7	647.4	38.9	-8.2	16.6	22.1	274.3	6.3	682.5	41.8	7.0	612.4	36.1
5	Todirești, SV	8.1	555.6	30.7	-7.5	18.4	24.3	232.9	7.8	561.5	31.5	8.3	549.8	30.0
6	Calafindești, SV	8.2	572.0	31.4	-7.2	18.6	24.2	238.4	7.9	580.7	32.5	8.6	563.2	30.3
7	Fetești, SV	8.5	539.7	29.1	-7.1	19.0	25.0	223.8	8.3	537.3	29.4	8.8	542.1	28.8
8	Zvoriștea, SV	8.2	572.0	31.4	-7.2	18.6	24.2	238.4	7.9	580.7	32.5	8.6	563.2	30.3
9	Zamostea, SV	8.2	572.0	31.4	-7.2	18.6	24.2	238.4	7.9	580.7	32.5	8.6	563.2	30.3
10	Probota, SV	8.7	523.7	28.0	-7.2	19.2	25.4	214.3	8.5	514.5	27.9	8.9	532.8	28.2
11	Dolhești, SV	8.7	523.7	28.0	-7.2	19.2	25.4	214.3	8.5	514.5	27.9	8.9	532.8	28.2
12	Liteni, SV	8.5	539.7	29.1	-7.1	19.0	25.0	223.8	8.3	537.3	29.4	8.8	542.1	28.8
13	Sirețel, IS	8.7	523.7	28.0	-7.2	19.2	25.4	214.3	8.5	514.5	27.9	8.9	532.8	28.2
14	Dolhasca, SV	8.7	523.7	28.0	-7.2	19.2	25.4	214.3	8.5	514.5	27.9	8.9	532.8	28.2
15	Preutești, SV	8.4	531.4	28.9	-7.5	18.8	25.0	221.0	8.2	525.6	28.9	8.6	537.2	28.9
16	Râșca, SV	7.6	556.0	31.5	-8.1	17.9	24.0	233.8	7.4	559.1	32.0	7.8	552.9	31.0
17	Mălini, SV	5.8	609.8	38.6	-9.2	15.7	21.4	258.4	5.6	636.4	40.7	6.0	583.1	36.5
18	Dumbrăveni, SV	7.6	556.0	31.5	-8.1	17.9	24.0	233.8	7.4	559.1	32.0	7.8	552.9	31.0
19	Buda, SV	7.6	556.0	31.5	-8.1	17.9	24.0	233.8	7.4	559.1	32.0	7.8	552.9	31.0
20	Negrileasa, SV	5.8	609.8	38.6	-9.2	15.7	21.4	258.4	5.6	636.4	40.7	6.0	583.1	36.5
21	Slatina, SV	5.8	609.8	38.6	-9.2	15.7	21.4	258.4	5.6	636.4	40.7	6.0	583.1	36.5
22	Voroneț, SV	5.8	609.8	38.6	-9.2	15.7	21.4	258.4	5.6	636.4	40.7	6.0	583.1	36.5
23	Mănăstirea Humorului, SV	7.2	593.8	34.6	-8.2	17.3	23.1	251.3	6.9	612.2	36.2	7.4	575.3	33.0
24	Păltinoasa, SV	7.2	593.8	34.6	-8.2	17.3	23.1	251.3	6.9	612.2	36.2	7.4	575.3	33.0
25	Frasin, SV	7.2	593.8	34.6	-8.2	17.3	23.1	251.3	6.9	612.2	36.2	7.4	575.3	33.0
26	Tătăruși, IS	7.9	536.8	30.0	-8.1	18.3	24.6	222.6	7.8	529.9	29.8	8.0	543.6	30.2
27	Todirești, IS	9.0	516.5	27.1	-6.8	19.7	25.9	207.5	8.8	505.7	26.9	9.3	527.4	27.3
28	Târgu Neamț, NT	7.9	536.8	30.0	-8.1	18.3	24.6	222.6	7.8	529.9	29.8	8.0	543.6	30.2
29	Văratec, NT	7.9	536.8	30.0	-8.1	18.3	24.6	222.6	7.8	529.9	29.8	8.0	543.6	30.2
30	Stănița, NT	9.1	521.6	27.3	-6.8	19.8	26.1	201.9	8.9	502.9	26.6	9.3	540.2	28.0
31	Gâcești, VS	9.3	517.3	26.8	-6.5	20.0	26.3	192.7	9.1	497.1	26.0	9.5	537.5	27.6
32	Oniceni, NT	9.1	521.6	27.3	-6.8	19.8	26.1	201.9	8.9	502.9	26.6	9.3	540.2	28.0
33	Poienari, NT	9.1	521.6	27.3	-6.8	19.8	26.1	201.9	8.9	502.9	26.6	9.3	540.2	28.0
34	Țibănești, VS	9.5	516.8	26.6	-6.4	20.2	26.5	195.7	9.2	501.0	26.0	9.7	532.7	27.1
35	Chițoc, VS	9.6	505.3	25.8	-6.1	20.5	26.6	181.6	9.4	487.8	25.1	9.8	522.8	26.4

36	Florești, VS	9.6	505.3	25.8	-6.1	20.5	26.6	181.6	9.4	487.8	25.1	9.8	522.8	26.4
37	Ivănești, VS	9.3	517.3	26.8	-6.5	20.0	26.3	192.7	9.1	497.1	26.0	9.5	537.5	27.6
38	Bonțești, VN	10.5	474.5	23.1	-5.8	21.4	27.5	171.5	10.5	464.6	22.7	10.5	484.3	23.6
39	Câmpuri, VN	6.7	559.2	33.5	-8.9	17.0	22.9	213.5	6.8	571.0	34.0	6.6	547.4	33.0
40	Tomești, IS	9.7	518.7	26.3	-6.1	20.6	26.9	195.1	9.4	514.4	26.5	10.0	523.0	26.2
41	Poieni, IS	9.7	518.7	26.3	-6.1	20.6	26.9	195.1	9.4	514.4	26.5	10.0	523.0	26.2
42	Pocreaca, IS	9.5	517.7	26.5	-6.2	20.3	26.6	191.5	9.3	505.9	26.2	9.7	529.5	26.8
43	Dobrovăț, IS	9.5	517.7	26.5	-6.2	20.3	26.6	191.5	9.3	505.9	26.2	9.7	529.5	26.8
44	Zebreni, IS	9.0	516.5	27.1	-6.8	19.7	25.9	207.5	8.8	505.7	26.9	9.3	527.4	27.3
45	Hârlău, IS	9.0	516.5	27.1	-6.8	19.7	25.9	207.5	8.8	505.7	26.9	9.3	527.4	27.3
46	Humosu, IS	8.7	523.7	28.0	-7.2	19.2	25.4	214.3	8.5	514.5	27.9	8.9	532.8	28.2
47	Poiana Deleni, IS	9.0	516.5	27.1	-6.8	19.7	25.9	207.5	8.8	505.7	26.9	9.3	527.4	27.3
48	Moțca, IS	8.7	523.7	28.0	-7.2	19.2	25.4	214.3	8.5	514.5	27.9	8.9	532.8	28.2
49	Iorcani, IS	8.7	523.7	28.0	-7.2	19.2	25.4	214.3	8.5	514.5	27.9	8.9	532.8	28.2
50	Crivești, IS	9.0	516.5	27.1	-6.8	19.7	25.9	207.5	8.8	505.7	26.9	9.3	527.4	27.3
51	Todirel, IS	9.7	518.7	26.3	-6.1	20.6	26.9	195.1	9.4	514.4	26.5	10.0	523.0	26.2
52	Fărcășești, IS	9.3	507.1	26.3	-6.7	20.0	26.3	202.7	9.1	490.8	25.7	9.5	523.4	26.9
53	Brăești, IS	9.6	504.8	25.8	-6.4	20.3	26.7	197.9	9.3	489.8	25.3	9.8	519.8	26.3
54	Popești, IS	9.6	509.8	26.0	-6.3	20.4	26.7	196.5	9.3	499.8	25.9	9.8	519.8	26.2
55	Mădârjac, IS	9.6	504.8	25.8	-6.4	20.3	26.7	197.9	9.3	489.8	25.3	9.8	519.8	26.3
56	Răducăneni, IS	9.7	515.5	26.2	-5.9	20.6	26.7	183.6	9.4	507.8	26.1	9.9	523.1	26.2
57	Poiana, BT	8.8	544.5	28.9	-6.8	19.3	25.2	224.4	8.5	542.8	29.4	9.2	546.2	28.5
58	Copălău, BT	9.4	509.5	26.3	-6.4	20.0	26.2	206.6	9.1	498.5	26.1	9.7	520.6	26.4
59	Oneaga, BT	9.4	509.5	26.3	-6.4	20.0	26.2	206.6	9.1	498.5	26.1	9.7	520.6	26.4
60	Tudora, BT	8.7	523.7	28.0	-7.2	19.2	25.4	214.3	8.5	514.5	27.9	8.9	532.8	28.2
61	Serafinești, BT	8.8	529.7	28.2	-6.9	19.3	25.4	217.1	8.5	522.6	28.3	9.1	536.8	28.2
62	Daneş, MS	8.5	538.5	29.1	-8.3	18.6	25.1	216.1	8.6	534.7	28.7	8.4	542.2	29.5

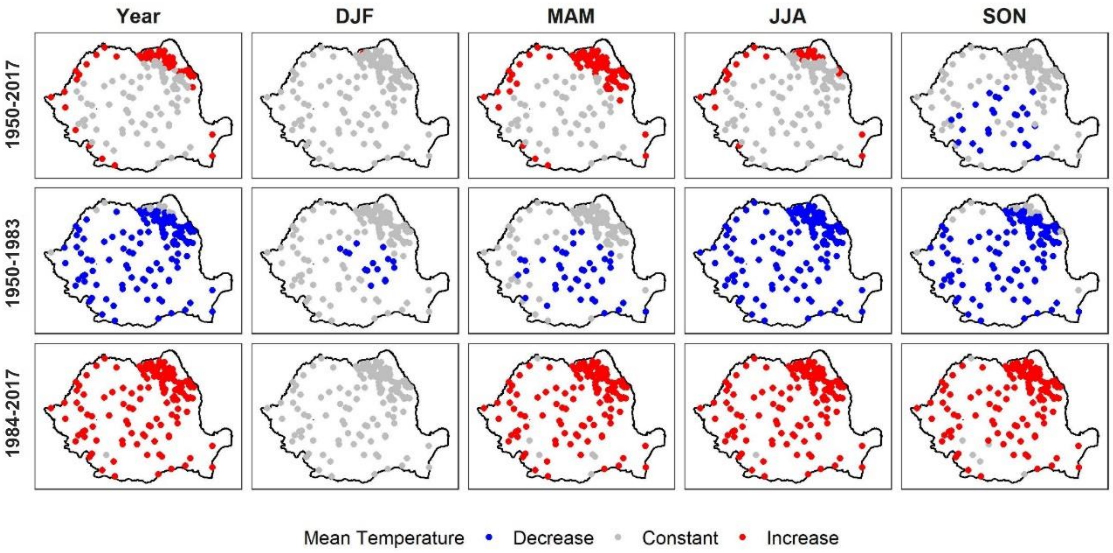

Figure S1. The trend of average annual and seasonal temperatures in the survey places between the years 1950-2017. DJF – December, January, February; MAM – March, April, May; JJA – June, Luly, August; SON – September, October, November

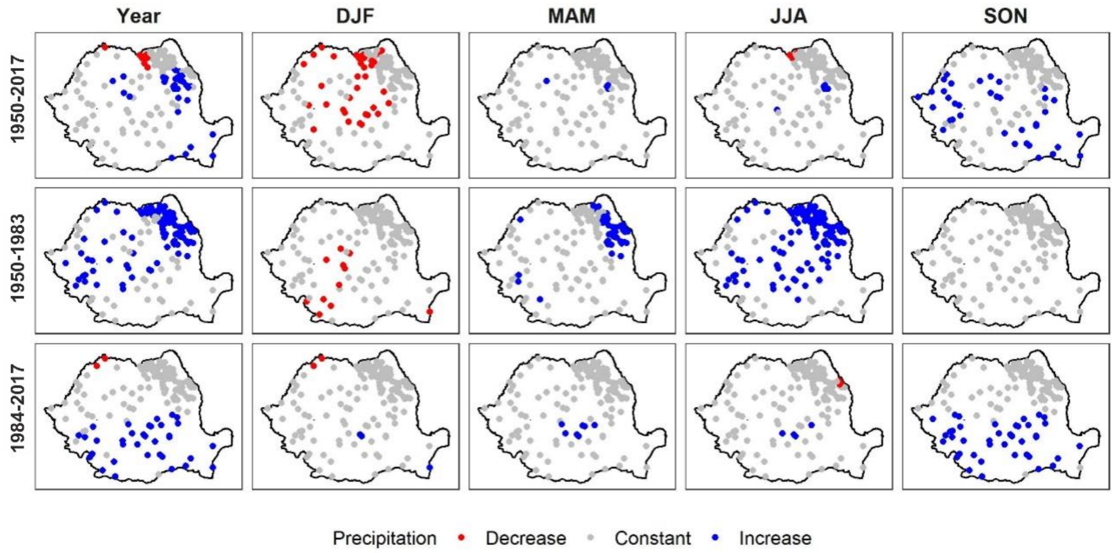

Figure S2. The trend of average annual and seasonal precipitation in the survey places between the years 1950-2017. DJF – December, January, February; MAM – March, April, May; JJA – June, Luly, August; SON – September, October, November

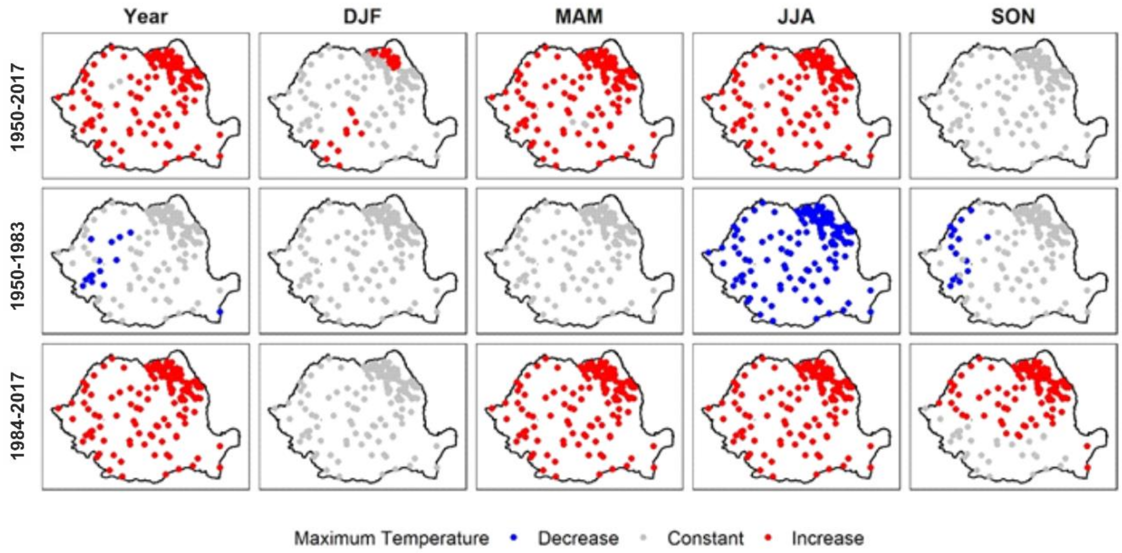

Figure S3. The trend of maximum annual and seasonal temperatures in the survey places during the years 1950-2017. DJF – December, January, February; MAM – March, April, May; JJA – June, Luly, August; SON – September, October, November

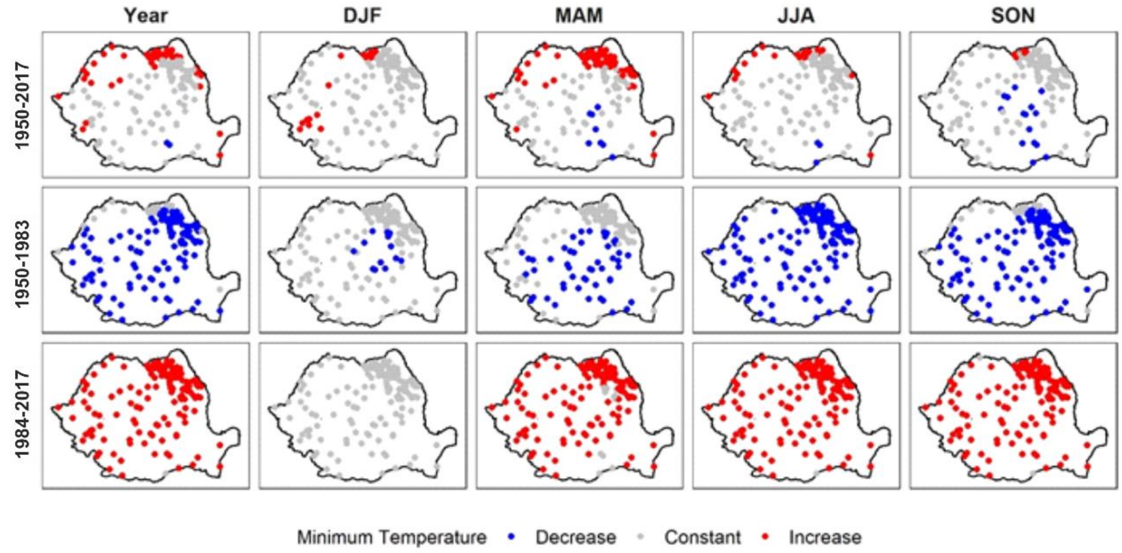

Figure S4. The trend of minimum annual and seasonal temperatures in the survey places during the years 1950-2017. DJF – December, January, February; MAM – March, April, May; JJA – June, Luly, August; SON – September, October, November

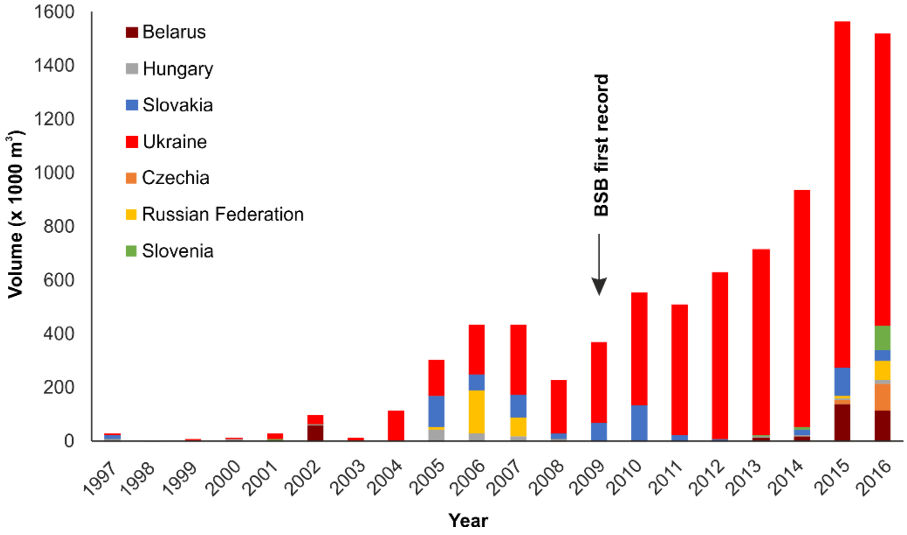

Figure S5. Romania's round wood import dynamics between 1997 and 2016, from the main European partners (Data source: FAO, 2018)
